# ERISA and the Failure of Employers to Perform Their Fiduciary Duties: Evidence from a Survey of Health Plan Administrators

**DOI:** 10.1017/jme.2025.10159

**Published:** 2026

**Authors:** Barak Richman, Amy Monahan, Jeffrey Pfeffer, Sara Singer

**Affiliations:** 1 School of Law, The George Washington University, United States; 2 Stanford School of Medicine, Clinical Excellence Research Center, United States; 3 University of Minnesota Law School, United States; 4 Stanford Graduate School of Business, United States

**Keywords:** erisa, employer-sponsored insurance, employer fiduciaries, employer health plans, group health plans

## Abstract

Employers purchase health benefits for more than 60% of the nonelderly population, making employers both important custodians of employee well-being and important actors in the health care ecosystem. Because employers typically have unilateral control over health and retirement benefits, the federal Employee Retirement Income Security Act (ERISA), enacted in 1974, imposes fiduciary obligations on employers when they manage or administer benefits. We provide evidence, from a novel survey of respondents who administer or oversee health benefits for their companies, that many employers appear to neglect even the most basic of their fiduciary obligations to their employees. This neglect may help explain the poor performance of employer plans in controlling costs and providing access to health care, and it suggests that many employers may be vulnerable to liability from ERISA lawsuits.

## I. Introduction

Employer-provided health plans are important financiers of American healthcare, spending approximately $1.5 trillion annually on employee health benefits^1^ and covering nearly 178 million individuals — 54% of the entire population and more than 80% of those with private health insurance.^2^ Although an important source of coverage, employer-provided health plans have exhibited a poor track record, allowing total healthcare costs to escalate while shifting cost burdens onto workers, thereby increasing medical debt and causing many individuals to forgo care.^3^ Policy analysts have lamented the lack of regulatory authority to reign in the wastefulness of commercial healthcare.^4^

Recently, however, scholars have identified the 50-year-old Employee Retirement Income Security Act (ERISA) as a potential tool that could improve employer offerings and, potentially, the nation’s health sector.^5^ Although historically ERISA has been more actively enforced against managers of retirement benefits than managers of health benefits, a small number of recent lawsuits have accused employers of violating ERISA by selecting plan administrators that charge above-market prices for covered plan benefits.^6^ This recent development in ERISA litigation and the academic literature raises the question of whether, and to what degree, employers are violating ERISA’s fiduciary duties and in danger of ERISA liability. For aspiring health policy reformers, these events additionally suggest that greater enforcement of ERISA’s fiduciary duties could improve the performance and value of employer-provided healthcare.

This paper offers a preliminary assessment of whether managers of employee health benefits appear to satisfy the legal requirements of ERISA. It first explains the fiduciary obligations ERISA imposes on employers and the minimum that those obligations require regarding health benefits. It then reviews recently reported evidence, based on a novel survey of benefits managers working inside companies that offer employer-sponsored insurance, that reveals what employers *actually do.* Or, as the results show, what employers *do not do.* It concludes that many sampled employers — at least one third and possibly more — may be failing to uphold ERISA’s obligation to act as a prudent fiduciary, based both on ERISA’s plain meaning and on the Supreme Court’s explication of the statute. In short, the data strongly suggest widespread noncompliance with ERISA requirements with respect to the oversight of health benefits.

The implications of these findings are twofold: first, many employers appear vulnerable to significant ERISA legal liability, something that has already occurred in class action suits in the domain of retirement benefits. And second, America’s employers are investing inadequate attention and effort into spending their workers’ health benefits dollars wisely, thereby failing to demand value from the entire health sector. Because employer-sponsored insurance covers most of the nation’s privately insured, this inattention allows market-wide inefficiencies to burden consumers.

## II. A Primer on ERISA’s Fiduciary Duties and Their Application to Health Benefits

After several companies severely mismanaged their employees’ pension benefits, Congress passed ERISA in 1974 to safeguard employee benefits.^7^ One of ERISA’s central features is the imposition of fiduciary duties on employers that manage employee benefit plans. These duties are derived from the common law of trusts, which provides that trustees owe a special duty of care to trust beneficiaries. While the trust analogy is easy to follow with respect to retirement plans, where money is set aside and invested for the benefit of plan participants, these fiduciary duties apply equally, if not obviously, to those who manage employer-provided health plans.

One complication of applying fiduciary duties in the context of employee benefit plans is that employers are not always acting as a fiduciary when they make decisions regarding employee benefits. Fiduciary duties do not apply when an employer makes decisions regarding plan formation, design, or termination.^8^ These decisions have been deemed to be non-fiduciary business decisions, labeled as “settlor functions” in ERISA parlance. With respect to health plans, this means that employers are not required to offer a plan and thus can freely make decisions regarding plan structure (such as setting deductibles and copayments) and covered benefits without being constrained by fiduciary duties.

Fiduciary standards apply when an employer is exercising “any discretionary authority or discretionary control respecting management” of an employee benefit plan or when an employer “has any discretionary authority or discretionary responsibility in the administration” of such a plan.^9^ The Department of Labor (DOL) has explicitly stated that, for health plans, “[h]iring a service provider in and of itself is a fiduciary function.”^10^ The term *service provider* is used expansively, and includes insurers, third party administrators, health care service providers, and pharmacy benefits managers. As a result, even employers that offer fully insured health plans, where many fiduciary functions are outsourced to an insurance company or other third-party administrator, retain fiduciary responsibility for the selection of the insurer.^11^

ERISA enumerates several fiduciary duties, two of which are relevant for our purposes. A fiduciary must discharge their duties “solely in the interest of plan participants and beneficiaries … for the exclusive purpose of providing benefits” (the duty of loyalty) and “with the care, skill, prudence, and diligence under the circumstances then prevailing that a prudent man acting in a like capacity and familiar with such matters would use in the conduct of an enterprise of a like character and with like aims” (the duty of prudence).^12^

The duty of loyalty is essentially a prohibition on self-dealing by fiduciaries. It prevents employers from using plan assets for their own purposes or gain, such as receiving kickbacks for placing plan investments or using plan assets to cover payroll expenses. For group health plans, the duty of loyalty has relatively limited applicability. For example, an employer’s decision to shift costs to employees in the form of an increased deductible would not be “solely in the interests of plan participants,” but such a decision is outside scrutiny because the structure of plan cost-sharing is not a fiduciary decision. Similarly, a decision to exclude coverage for a particular type of medical treatment may not be in participants’ interests, but it too is not a fiduciary decision because it regards plan design. The most common scenario seen in case law with respect to the duty of loyalty as applied to health plans is when an employer withholds funds from employee wages to pay for health insurance premiums but then uses those funds for unrelated business purposes, rather than forwarding such amounts to the insurer on a timely basis.^13^

The duty of prudence has far greater implications for health plan sponsors. The duty of prudence is a processed-based duty that requires careful investigation and evaluation when exercising discretion in plan management or administration. Compliance can be shown by having a robust decision-making process that ensures that fiduciaries, i.e., employers, have access to and consult relevant information necessary to make plan decisions, and that such decisions are carefully considered.^14^ Employers do not need to reach a single, objectively correct decision to satisfy the duty. Instead, as the Supreme Court has acknowledged, there are typically a “range of reasonable judgments a fiduciary may make based on her experience and expertise.”^15^

Although there is no rigid process that employers must follow to satisfy this duty of prudence, fifty years of ERISA case law and regulatory guidance has produced useful standards that employers must satisfy. We highlight three below.

### Making Informed Decisions When Selecting Plan Service Providers

One of the most important fiduciary decisions an employer that offers a group health plan makes is selecting the plan’s service providers, including third-party administrators, insurers, pharmacy benefits managers, and utilization reviewers.^16^ Not all health plans separately contract with each of these types of vendors, but the choice of either an insurer or a third-party administrator is nearly universal. Focusing just on that primary selection, it is clear that a fully informed decision-making process is not terribly easy to achieve. According to guidance from the DOL, the employer must evaluate

the scope of choices and qualifications of medical providers and specialists available to participants, ease of access to medical providers, ease of access to information concerning the operations of the health care provider, the extent to which internal procedures provide for timely consideration and resolution of patient questions and complaints, the extent to which internal procedures provide for the confidentiality of patient records, enrollee satisfaction statistics, and rating or accreditation of health care service providers by independent services or state agencies.^17^

Given the complexity of this task, many employers seek outside consultants or brokers to guide them through their decision-making. Although fiduciaries are encouraged, and perhaps expected, to consult outside experts to inform their decisions, reliance on experts does not discharge employers’ fiduciary duties; employers retain a fiduciary duty to exercise independent judgment. As the Court of Appeals for the Third Circuit explained,

While we would encourage fiduciaries to retain the services of consultants when they need outside assistance to make prudent investments and do not expect fiduciaries to duplicate their advisers’ investigative efforts, we believe that ERISA’s duty to investigate requires fiduciaries to review the data a consultant gathers, to assess its significance and to supplement it where necessary.^18^

As a result, employers’ obligations persist even when employers retain experts. This fiduciary obligation cannot be outsourced.

### Reasonable Compensation of Service Providers

In selecting a plan service provider, the employer should not only gather all relevant information and consult experts where necessary, but must also ensure that the fees charged are “reasonable in light of the services provided.”^19^ In making this determination, the DOL has advised that the quality of the services offered is a relevant factor in this determination, and that failure to take into account quality when selecting a service provider and determining whether its fees are reasonable would constitute a breach of fiduciary duty.^20^

Historically, an employer’s determination of whether health plan fees are reasonable has been difficult for two primary reasons. First, evaluating the “quality” of services is difficult because the services provided include not only the administrative services offered by the insurer or TPA (which would include things like claims processing accuracy and telephone wait times), but also the underlying quality of the medical care delivered by the insurer’s or TPA’s network of providers.

Second, employers historically have had very little information available to them about relative prices of covered benefits for which they are contracting, with many insurers and TPAs treating their negotiated provider rates as confidential and proprietary. Similarly, employers historically have not received adequate disclosure of the various forms of indirect compensation that their service providers expect to receive, whether through various commissions or services that are provided by related parties.

Recent legal changes have started to address some of these issues, although the market is still far from transparent. Notably, as of January 1, 2021, hospitals are required to post payer-specific pricing information online in a machine-readable format for all items and services the hospital provides. These data, where available,^21^ should allow employers to determine how the hospital prices negotiated on their behalf by their insurer or TPA compare to other private payers, and to the available cash pay price. In addition, the Consolidated Appropriations Act of 2021 requires brokers and consultants to disclose to health plan fiduciaries all direct and indirect compensation expected to be received from a proposed contract.^22^

Thus, even though the obligation to gather adequate information to ensure that fees are “reasonable in light of the services provided” has historically been difficult to enforce, recent regulatory and market developments have made such an inquiry more feasible. As price and quality information become increasingly obtainable, expectations sharpen for employers to prudently consult the data that are available and determine, based on that information, whether the fees charged by service providers are reasonable in light of the services provided.

### Ongoing Monitoring of Plan Service Providers

A prudent fiduciary not only makes well-informed decisions when selecting a plan service provider and makes sure such providers are paid only reasonable compensation, but it also monitors that provider’s performance at “reasonable intervals.”^23^ It is notable that in this era of severe ideological disagreement among the Supreme Court, the Justices have been undivided in reiterating the ongoing obligations that employers have as fiduciaries. In two unanimous opinions, in 2015 and 2022, the Court emphasized that employers have a “continuing duty” to monitor retirement plan investments.^24^ It is not enough to make a prudent decision at the outset, but a fiduciary must monitor whether the choices previously made continue to satisfy the duty of prudence; it is no defense to argue that an imprudent feature of a benefits plan is a legacy of an earlier regime. The duty of prudence requires steady stewardship and regular oversight.

In sum, an employer that offers health benefits to employees must select plan service providers only after completing an appropriate amount of research and deliberation, including consulting with experts as appropriate. This review of potential third-party administrators or insurers for the plan should include not only an evaluation of the administrator’s customer service and administrative skills and capabilities, but also the adequacy, quality, and cost of the administrator’s network of providers. Finally, once an administrator is selected, the employer has a continuing fiduciary duty to monitor its performance along these same metrics.

## III. What Employers Actually Do — Results from an Original Survey

With these well-established fiduciary responsibilities under ERISA as background, we sought to discover what employers actually did for health benefits oversight and management. We conducted a novel survey in 2022, administered by SSRS, a well-known survey research firm, to a nationally representative sample of employee health benefits managers at companies with at least 50 employees. A total of 221 firms completed responses, constituting a 22% response rate, with statistically insignificant differences between respondents and nonrespondents regarding firm size, geographic location, and industry.^25^

Our survey included four categories of questions. One set asked employers how they shopped for, bargained with, and contracted with health plan administrators. We found that 66% of companies said they requested offers from multiple health benefit administrators, 62% said they conducted benchmark research to compare their plan with the plans of similar companies, and 48% said they negotiated price directly with the health benefit administrator (see Appendix Q21).

A second set of questions asked how employers assessed the value and performance of the health plans with which they contract on behalf of their employees. Very few employers scrutinized the dimensions of their plan’s performance in detail. When asked about 15 health benefits performance elements that employers could use to measure and manage health plans, employers reported measuring an average of only 3.5 elements (23%), with 43% of employers measuring fewer than 5 of the 15 and only 4% of employers measuring more than 10 (see Appendix Q19).^26^ Employers reported someone was responsible for managing an average of 36% of the same 15 health benefits performance elements, meaning that 64% were not managed (see Appendix Q20).

A third set of questions addressed how employers sought to improve the value of the plans offered to their workforce. Although many employers monitor aspects of health benefits spending, many failed to use specific strategies that can reduce the costs and increase the value of their health plans. Only 18% of employers contracted directly with service providers, and only 10% of employers offered a narrow network health plan option, one that directs insureds towards doctors and hospitals with lower-than-average prices but at least average quality (see Appendix Q21). More generally, very few employers invested in trying to reduce health benefits spending, the savings of which (according to economic theory) would naturally accrue to their employees. When asked about seven common strategies to reduce health benefits spending — two of which involved cost shifting to insureds and five of which involved changes to plan design and employee incentives — cost shifting proved more popular. On average, only 18% of respondents said their companies used these plan design strategies and 14% reported using financial incentives to reduce spending. Meanwhile, 32% said their companies relied on cost shifting to their employees (see Appendix Q22).

A final set of questions asked whether employers solicited the opinions and experiences of their employees, which may be the simplest way to improve their health benefits offerings and monitor service provider performance. Almost 2 out of 5 (39%) companies either *never* requested feedback from their employees about their health benefits, couldn’t recall ever having done so, or refused to answer the question (see Appendix Q26). Even fewer firms tracked employee opinions about either company health benefits or the health benefits administrator (20%), the time employees spent having questions answered from their health benefits administrator (6%), or how often employees postponed filling a prescription, visiting a doctor, or having a medical procedure because of the cost of care (<5%) (see Appendix Q19). Additionally, very few employers monitored how their employees engaged with the plan’s review processes: only 20% tracked the number of claims initially or ultimately denied and 15% tracked the number of grievances or appeals filed (see Appendix Q19).

## IV. The Legal Implications of Employer Nonfeasance

Although ERISA’s fiduciary duties offer only general standards and are applied contextually, and thus cannot easily be compared with survey data, these survey results strongly suggest many employers are failing to comply with even basic fiduciary duties and therefore could be subject to legal liability. The most likely source of liability stems from our first finding, that many firms appear to have failed to adequately invest in a robust shopping process for insurers or TPAs. A second, less certain but nonetheless strong possibility of ERISA liability follows from our second finding, that employers do not rigorously monitor their health plan offerings each year. And finally, though there are few substantive requirements for minimal quality and value that employers are required to offer, it appears that many employers might risk ERISA liability for failing to invest in rudimentary efforts to ensure that the compensation paid to plan service providers is reasonable in light of the services provided.

Before examining each of these potential sources of liability, it is first helpful to understand the basic structure of ERISA’s remedial scheme for breaches of fiduciary duty. ERISA authorizes plan participants, beneficiaries, and the Secretary of Labor to commence a civil action to “make good” any losses to the plan that result from a breach of fiduciary duty and to receive any appropriate equitable relief.^27^ With respect to making good losses to the plan, a claimant must generally establish the monetary harm suffered by the plan as a result of the fiduciary breach in order to receive a monetary award. For example, with respect to the duty of prudence, it is not enough to establish that the fiduciary failed to undertake a prudent decision-making process with respect to selecting a plan service provider; the claimant must also establish that the plan (and by extension, its participants) were concretely harmed thereby. If an imprudent fiduciary lucks into making a reasonable decision, there is no loss to the plan and therefore no recovery. Equitable relief is potentially more forgiving, as it could be used to require the employer to reform its decision-making processes, but plaintiffs seeking only equitable relief may lack constitutional standing if they cannot allege a concrete, particularized harm.^28^

### Failure to Proactively Shop for Third-Party Administrators, Insurers, or Pharmacy Benefits Managers

Our survey results show a surprising lack of active shopping for core plan service providers such as TPAs, insurers, or pharmacy benefits managers, despite the fact that the duty of prudence clearly requires fiduciaries to comparison shop.^29^ According to the DOL, before selecting a health plan service provider the fiduciary should

[get] information from more than one provider; [compare] firms based on the same information, such as services offered, experience, costs, etc.…[consider] whether fee charged to a plan…are ‘reasonable’” and “[document] its selection…process.^30^

Yet our results indicate that 33% of employers do not request offers from multiple contractors and 37% do not compare their plan’s offered price with those of others. On this basis alone, we find that more than one-third of surveyed employers are likely in violation of ERISA.

Comparison shopping and price negotiation may be difficult due to provider consolidation, but this alone does not relieve employers of their duty to make informed purchasing decisions. Moreover, nearly all metropolitan statistical areas contain some insurer competition in the employer market,^31^ and failing to actively shop for health benefits increases costs — for healthcare services, pharmacy benefits, administrative costs, and the like — for both employers and employees.

Our survey results conform with popular reports that many employers fail to assert themselves as informed consumers.^32^ Many employers reportedly select plan service providers who negotiate prices that *exceed* cash prices, those paid by customers paying in cash without any negotiating leverage.^33^ A prudent fiduciary would compare the prices offered by competing vendors and, decidedly, would not agree to a contract that charges more, in aggregate, than the cash price for covered services.

### Failure to Monitor Service Providers

According to DOL guidance, employers sponsoring health plans should “establish a formal review process and follow it at reasonable intervals to decide if it wants to continue using the current service providers or look for replacements.”^34^ As part of this review, plan fiduciaries should review the service provider’s performance, check the fees charged, review the provider’s policies and practices, and “follow-up on participant complaints.”^35^

In our survey, nearly all respondents reported reviewing their health plan annually, thus appearing to satisfy the most basic duty to monitor, but many of these annual reviews may fall short of fiduciary standards. Very few employers employed industry-standard performance measures, and most employers did very little to measure employee experience, meaning they certainly could not monitor it. Although ERISA does not require employers to ensure employee contentment, employers are under a duty to monitor service providers at regular intervals, and employee experience and access to care are critical quality factors.

### Failure to Ensure No More Than Reasonable Compensation is Paid for Plan Services

Both prior failures contribute to the third — a failure to ensure that compensation paid to service providers is reasonable in light of the services provided. If the plan fiduciary is neither comparison shopping for services nor monitoring performance, it is nearly impossible to ensure that this fiduciary requirement is satisfied.

Our findings help explain why employer-sponsored insurance has grown in cost, reducing employee take-home pay. In 2023, the average annual premium for employer plans for family coverage was $23,968 (equal to more than 25% of the median family household income of $95,450) and $8,435 for individual coverage (equal to nearly 20% of median non-family household income of $45,440), amounts that have further grown as of 2024.^36^ These premiums, and the underlying per-enrollee spending of such plans, have risen far faster than baseline inflation.^37^ Employer plan reimbursement rates have also grown at a much higher rate than Medicare prices. From 1996 to 2001, private insurers paid hospitals approximately 10% more than Medicare, but in 2012 private plans paid 75% more,^38^ and the most recent data suggest that private plans now pay 224% of what Medicare pays hospitals for identical services.^39^ At least some of these inflated costs could be attributed to poor negotiations by employers and the insurers they hired to administer their plans.

Additionally, employers have shifted increased costs to employees in the form of higher copayments, coinsurance, and deductibles. Most workers with employer coverage are currently in a plan with an annual deductible, which on average is $1,787 and for 32% of covered workers is greater than $2,000.^40^ Both the percentage of workers in plans with an annual deductible and the average dollar amount of such deductibles have grown significantly in recent years. From 2006 to 2022, deductibles have increased 162%, whereas inflation was 20% and workers’ earnings grew by 26%.^41^

### The Scope of Potential Liability

While our survey results suggest widespread failure to prudently select and monitor plan service providers, the scope of potential legal liability is difficult to determine with specificity, given that ultimate liability would depend on whether prudent fiduciaries acting in similar circumstances would have been able to secure a more favorable arrangement. Market conditions such as provider consolidation can therefore act as de facto liability shields even for clearly imprudent decision-makers. Perhaps not surprisingly, the initial lawsuits filed in this area have targeted the selection of pharmacy benefit managers, where it is relatively easy for potential plaintiffs and their attorneys to determine the extent to which the prices charged by PBMs exceed other prices in the market. These cases are in the early stages of litigation and as a result many questions remain about the level of proof necessary to establish that a breach of fiduciary duty has resulted in a loss to plan, as well as the remedies that would be available in the event both a breach and a resulting loss have been established.^42^

## V. Paths Forward

Our survey data suggest that many employers may be failing to comply with their legal duties and may face significant liability as a result. While our findings offer some clear lessons for employers, they also suggest that policymakers and employee advocates who are eager for health plan improvements could use ERISA to nudge employers toward better decision-making.

### What Employers Should Do

There clearly is much that employers could do voluntarily to avoid ERISA liability. First, they can and should comply with ERISA’s most rudimentary and self-evident fiduciary duties. Employers selecting an insurer, TPA, or pharmacy benefits manager should solicit multiple bids from service providers, make sure they understand how those service providers are receiving compensation under the proposed contract, evaluate both the administrative and clinical quality of services being offered, and compare pricing. Where an employer lacks expertise to make these determinations, an independent expert should be retained to assist with service provider selection. Once a service provider is selected, the employer should monitor its performance on an ongoing basis and solicit feedback from employees at least annually.

Additionally, employers should be attentive to the contracts they sign. For example, employers might propose contractual performance guarantees, with automatic penalties for the service provider in the event those guarantees are not met, or contractually require service providers to share claims information with the employer on a deidentified basis. Employers that secure such data can monitor healthcare prices and the health status of their employee populations, as many large employers have shown by securing and analyzing such data through integrated warehouses.^43^

The ability to negotiate contract terms will vary with employer size and market characteristics, and smaller employers may struggle to implement certain best practices, but abundant examples illustrate how larger employers have successfully bargained for novel contract terms, such as Medicare reference pricing in lieu of negotiated rates,^44^ and even where employers have directly contracted with health systems in order to negotiate prices themselves.^45^ Fiduciary duty expectations always vary by context, but even small employers can ensure that they pay no more than reasonable compensation for services, not only through comparison shopping, but also through novel strategies such as purchasing cooperatives that attain economies of scale. For small employers worried about their ability to comply with the fiduciary duties that apply to service provider selection and monitoring, other avenues are available to help employees afford and access health care that require a lower level of fiduciary oversight, such as providing employees with a tax-free employer contribution to purchase individual health insurance coverage on an exchange.

Additional lessons come from organizational behavior principles. Our survey and other studies show that health benefits administration is an afterthought for many employers. Even though there is ample evidence that employee health and well-being positively affect productivity and negatively affect voluntary turnover, the effectiveness of benefits administration is not much of a strategic priority. Assigning benefits oversight to line executives with some degree of influence would be an important step toward improving stewardship of this important function.

### What Policymakers and Employee Advocates Should Consider

If employers fail to act voluntarily, there are opportunities for policymakers, advocates, and employees to induce employers to take needed action.

It is worth recalling that employers became better stewards of retirement benefits when they faced (expensive) class action suits demanding that they do a better job of negotiating administrative fees and providing lower-cost investment options. In 2019, it was reported that “corporations have paid out $6.2 billion in class-action lawsuits in which employees claimed that the companies acted improperly in the administration of their 401(k) or defined benefits pension or retiree plans,”^46^ a number that has only grown as 401(k) lawsuits continue to proliferate.^47^

Similar lawsuits have been initiated that target employer management of health benefits, and some of the same law firms that were active in the retirement benefits litigation are considering medical benefits. As these suits both increase in number and gain broader attention, compliance resources will rise, and employers will be more likely to devote resources to ensuring their legal obligations are satisfied.

Additionally, the DOL has authority to bring enforcement actions to ensure that employers comply with ERISA’s fiduciary duties and could promulgate regulations to bring greater clarity to the contours of such duties. While one may be skeptical that the current administration would invest additional resources in such guidance and enforcement, it is worth noting that the current hospital price transparency rules were promulgated during the first Trump administration. To the extent that regulation and enforcement of ERISA’s existing fiduciary duties may be seen as part of an effort to enhance market competition in healthcare, greater DOL involvement may be more politically feasible.

## VI. Conclusion

Our findings suggest that a nontrivial percent of surveyed employers, at least one-third, does *not* satisfy even the most basic of ERISA’s fiduciary duties. This failure exposes these employers and many like them to potential liability under ERISA, but more importantly, it has led to unnecessarily high healthcare expenditures and a less healthy and satisfied workforce. And because employers play outsized roles in purchasing healthcare for Americans, their inattention systemically permits inefficiencies across the broader national market.

However, it seems that there are signs of change. Whether employers like it or not, they will soon need to answer to ERISA’s demands and become better stewards of their employees’ healthcare dollars if they remain committed to sponsoring employer-sponsored insurance.

## Supporting information

Richman et al. supplementary material Richman et al. supplementary material

## Supplementary material

The supplementary material for this article can be found at http://doi.org/10.1017/jme.2025.10159.

## Note

Sara Singer’s work on this manuscript was supported by Grant 80330 from the Robert Wood Johnson Foundation. Barak Richman has received funding from the Arnold Foundation and CERC, Stanford School of Medicine. The other authors have no funding or conflicts of interest to report.
